# Isoform-Specific Potentiation of Stem and Progenitor Cell Engraftment by *AML1/RUNX1*

**DOI:** 10.1371/journal.pmed.0040172

**Published:** 2007-05-15

**Authors:** Shinobu Tsuzuki, Dengli Hong, Rajeev Gupta, Keitaro Matsuo, Masao Seto, Tariq Enver

**Affiliations:** 1 Division of Molecular Medicine, Aichi Cancer Center Research Institute, Nagoya, Japan; 2 Molecular Haematology Unit, Weatherall Institute of Molecular Medicine, University of Oxford, Oxford, United Kingdom; 3 Division of Epidemiology and Prevention, Aichi Cancer Center Research Institute, Nagoya, Japan; Erasmus University, The Netherlands

## Abstract

**Background:**

*AML1/RUNX1* is the most frequently mutated gene in leukaemia and is central to the normal biology of hematopoietic stem and progenitor cells. However, the role of different AML1 isoforms within these primitive compartments is unclear. Here we investigate whether altering relative expression of AML1 isoforms impacts the balance between cell self-renewal and differentiation in vitro and in vivo.

**Methods and Findings:**

The human *AML1a* isoform encodes a truncated molecule with DNA-binding but no transactivation capacity. We used a retrovirus-based approach to transduce *AML1a* into primitive haematopoietic cells isolated from the mouse. We observed that enforced AML1a expression increased the competitive engraftment potential of murine long-term reconstituting stem cells with the proportion of AML1a-expressing cells increasing over time in both primary and secondary recipients. Furthermore, AML1a expression dramatically increased primitive and committed progenitor activity in engrafted animals as assessed by long-term culture, cobblestone formation, and colony assays. In contrast, expression of the full-length isoform AML1b abrogated engraftment potential. In vitro, AML1b promoted differentiation while AML1a promoted proliferation of progenitors capable of short-term lymphomyeloid engraftment. Consistent with these findings, the relative abundance of *AML1a* was highest in the primitive stem/progenitor compartment of human cord blood, and forced expression of *AML1a* in these cells enhanced maintenance of primitive potential both in vitro and in vivo.

**Conclusions:**

These data demonstrate that the “a” isoform of AML1 has the capacity to potentiate stem and progenitor cell engraftment, both of which are required for successful clinical transplantation. This activity is consistent with its expression pattern in both normal and leukaemic cells. Manipulating the balance of AML1 isoform expression may offer novel therapeutic strategies, exploitable in the contexts of leukaemia and also in cord blood transplantation in adults, in whom stem and progenitor cell numbers are often limiting.

## Abstract 2

The truncated "a" isoform of AML1 is shown to have the capacity to potentiate stem and progenitor cell engraftment, both of which are required for successful clinical transplantation.

## Editors' Summary

**Background.:**

Blood contains red blood cells (which carry oxygen round the body), platelets (which help the blood to clot), and white blood cells (which fight off infections). All these cells, which are regularly replaced, are derived from hematopoietic stem cells, blood-forming cells present in the bone marrow. Like all stem cells, hematopoietic stem cells self-renew (reproduce themselves) and produce committed progenitor cells, which develop into mature blood cells in a process called hematopoiesis. Many proteins control hematopoiesis, some of which are called transcription factors; these factors bind to DNA through their DNA-binding domain and then control the expression of genes (that is, how DNA is turned into proteins) through particular parts of the protein (their transcription regulatory domains). An important hematopoietic transcription factor is AML1—a protein first identified because of its involvement in acute myelogenous leukemia (AML, a form of blood cancer). Mutations (changes) in the *AML1* gene are now known to be present in other types of leukemia, which are often characterized by overproliferation of immature blood cells.

**Why Was This Study Done?:**

Because of AML1′s crucial role in hematopoiesis, knowing more about which genes it regulates and how its activity is regulated could provide clues to treating leukemia and to improving hematopoietic cell transplantation. Many cancer treatments destroy hematopoietic stem cells, leaving patients vulnerable to infection. Transplants of bone marrow or cord blood (the cord that links mother and baby during pregnancy contains peripheral blood stem cells) can replace the missing cells, but cord blood in particular often contains insufficient stem cells for successful transplantation. It would be useful, therefore, to expand the stem cell content of these tissues before transplantation. In this study, the researchers investigated the effect of AML1 on self-renewal and differentiation of hematopoietic stem and progenitor cells in the laboratory (in vitro) and in animals (in vivo). In particular, they have asked how two isoforms (closely related versions) of AML1 affect the ability of these cells to grow and differentiate (engraft) in mice after transplantation.

**What Did the Researchers Do and Find?:**

The researchers artificially expressed AML1a and AML1b (both isoforms contain a DNA binding domain, but only AML1b has transcription regulatory domains) in mouse hematopoietic stem and progenitor cells and then tested the cells' ability to engraft in mice. AML1a-expressing cells engrafted better than unaltered cells and outgrew unaltered cells when transplanted as a mixture. AML1b-expressing cells, however, did not engraft. In vitro, AML1a-expressing cells grew more than AML1b-expressing cells, whereas differentiation was promoted in AML1b-expressing cells. To investigate whether the isoforms have the same effects in human cells, the researchers measured the amount of AML1a and AML1b mRNA (the template for protein production) made by progenitor cells in human cord blood. Although AML1b (together with AML1c, an isoform with similar characteristics) mRNA predominated in all the progenitor cell types, the relative abundance of AML1a was greatest in the stem and progenitor cells. Furthermore, forced expression of AML1a in these cells improved their ability to divide in vitro and to engraft in mice.

**What Do These Findings Mean?:**

These findings indicate that AML1a expression increases the self-renewal capacity of hematopoietic stem and progenitor cells and consequently improves their ability to engraft in mice, whereas AML1b expression encourages the differentiation of these cell types. These activities are consistent with the expression patterns of the two isoforms in normal hematopoietic cells and in leukemic cells—the mutated AML made by many leukemic cells resembles AML1a. Because the AML1 isoforms were expressed at higher than normal levels in these experiments, the physiological relevance of these findings needs to be confirmed by showing that normal levels of AML1a and AML1b produce similar results. Nevertheless, these results suggest that manipulating the balance of AML1 isoforms made by hematopoietic cells might be useful clinically. In leukemia, a shift toward AML1b expression might slow the proliferation of leukemic cells and encourage their differentiation. Conversely, in cord blood transplantation, a shift toward AML1a expression might improve patient outcomes by expanding the stem and progenitor cell populations.

**Additional Information.:**

Please access these Web sites via the online version of this summary at http://dx.doi.org/10.1371/journal.pmed.0040172.

- Wikipedia has pages on hematopoiesis and hematopoietic stem cells (note: Wikipedia is a free online encyclopedia that anyone can edit; available in several languages)
- The US National Cancer Institute has a fact sheet on bone marrow and peripheral blood stem cell transplantation (in English and Spanish) and information for patients and professionals on leukemia (in English)
- The American Society of Hematology provides patient information about blood diseases, including information on bone marrow and stem cell transplantation

## Introduction

*AML1* (also termed *RUNX1* or *CBFA2*), which encodes a transcription factor, was originally identified as a component of the fusion gene generated as a consequence of the t(8; 21) chromosomal translocation characteristic of a subset of acute myelogenous leukaemia [1]. Subsequently, mutations in the *AML1* gene were shown to be associated with a number of malignant and premalignant conditions including acute myelogenous leukaemia , childhood acute lymphocytic leukaemia, familial platelet disorder, and myelodysplastic syndromes. *AML1* is thus the most frequently mutated gene in human leukaemia [2,3]. *Aml1* expression marks long-term repopulating hematopoietic stem cells (HSCs) in the midgestation mouse embryo [4], and gene targeting experiments have unequivocally shown *Aml1* to be essential for the development of the hematopoietic system; *Aml1*-null mice die in utero of haemorrhage and lack adult-type hematopoiesis because of a failure in the generation of definitive HSCs [5,6]. In contrast, conditional gene-targeting experiments have revealed that *Aml1* is dispensable for maintenance of adult HSCs. *Aml1* deficiency in adult stages is accompanied by increases in myeloid progenitors and phenotypically defined HSCs, as well as impaired development of lymphoid and megakaryocytic cells [7–9].

While *AML1* is known to regulate the expression of many hematopoietic genes, including those encoding cytokines and their receptors as well as other transcription factors, the full spectrum of its target genes in different cell types in normal hematopoiesis, or *AML1*-associated leukaemia, remains unknown [10,11]. Furthermore, the manner in which *AML1* is itself regulated at the level of transcription and post-transcriptional processing is largely not understood [12]. Addressing these issues will be essential to understanding how *AML1* plays such a crucial role in both normal and leukaemic hematopoiesis. Potentially confounding factors in these endeavours are the large size of the *AML1* locus, the complexity of its putative regulatory elements, and the prevalence within the *AML1* gene of extensive alternative splicing [13–15].

Evidence is accumulating that isoforms of key hematopoietic transcription factors can play both distinct and key regulatory roles in hematopoiesis. A case in point is GATA1, which regulates the differentiation of erythroid and megakaryocytic cells [16]. Full-length GATA1 is composed of an amino-terminal transactivation domain as well as two zinc fingers that mediate its binding to DNA. However, an isoform lacking the transactivation domain is also expressed. This short form is impaired in its ability to promote differentiation and is implicated in abnormal hematopoiesis associated with Down syndrome [17,18]. Similarly, truncated forms of SCL/TAL1 favour erythroid lineage development, whereas full-length forms enhance the development of the megakaryocytic lineage [19]. An alternatively spliced isoform of TEL/ETV6 has recently been shown to inhibit, in a dominant-negative manner, the activity of full-length TEL and thereby negatively regulate the TEL-mediated erythroid differentiation of mouse erythroleukaemia cells [20]. Finally, enforced expression of an isoform of IKAROS/IKZF (IKAROS 6), which lacks the DNA-binding domain, in human CD34^+^ cells limits B cell development [21]. IKAROS isoforms are also implicated in the development of leukaemia [22–24].

The human *AML1* gene generates three alternatively spliced variants, *AML1a, AML1b,* and *AML1c* [25]. AML1b and AML1c both possess the DNA-binding region (runt domain) at the amino-terminal end as well as the carboxyl-terminal transcriptional regulatory domains; AML1b and AML1c are therefore considered to be broadly similar in function. In contrast, AML1a retains the DNA-binding domain, but lacks the transcriptional regulatory domains. AML1a is thus considered a potential functional antagonist of AML1b and AML1c [25]. The opposing functions of AML1a and AML1b have been shown in myeloid-committed cell lines with respect to their granulocyte colony-stimulating factor (G-CSF)–induced differentiation programmes; ectopically expressed AML1a blocks granulocytic differentiation, and this blockade is released by enforced expression of AML1b. Differentiation arrest in this cell-line model is accompanied by uncompromised growth under culture conditions that normally induce differentiation in the parental cell line [26]. *AML1a* transcripts have also been noted in selected leukaemia samples, as have mutant truncated forms of the AML1 protein that structurally resemble AML1a [26–29]. However, little is known about the potentially differential roles of AML1 isoforms in regulating normal or leukaemic cell fates in the stem and progenitor compartment of hematopoiesis. To address these questions, we expressed *AML1a* and *AML1b* in primary murine bone marrow (BM)-derived hematopoietic stem/progenitor cells and analysed the resulting effects on hematopoiesis both in vitro and in vivo. We then performed similar experiments in the corresponding human compartments. Such studies may lead to new strategies for the manipulation of haematopoietic progenitors and inform future treatments for haematological malignancies

## Methods

### Generation of Retroviruses

cDNAs for *AML1a* and *AML1b* [25] (generous gifts of Misao Ohki, National Cancer Center, http://www.ncc.go.jp) tagged with the FLAG-epitope at the amino terminal were cloned upstream of the internal ribosome entry site (IRES) element into the EcoRI/XhoI restriction site of the murine stem cell virus–internal ribosome entry site–green fluorescent protein (GFP) vector (MSCV-IRES-GFP) [30]. The MSCV-IRES-human CD4 vector that expresses an extracellular portion of the human CD4 molecule was generously provided by Jason G. Cyster (University of California San Francisco, http://www.ucsf.edu) [31]. Retrovirus vector plasmids were transiently transfected into Phoenix-Eco packaging cells (obtained from American Type Culture Collection, http://www.atcc.org) by a standard calcium phosphate precipitation method. Virus supernatants were then concentrated by centrifugation before use in infecting cells [32].

### Retroviral Infection of Mouse BM Cells and Transplantation

BM cells were harvested from tibiae and femora of BALB/c mice 4 d after intravenous administration of 5-fluorouracil (5-FU) (150 mg/kg) and treated with ACK (0.15 M NH_4_Cl, 1.0 mM KHCO_3_, 0.1 mM EDTA) to lyse red blood cells. BM cells were then prestimulated with stem cell factor (SCF) (100 ng/ml) + interleukin (IL) 3 (IL3) (10 ng/ml) + IL6 (6 ng/ml) (Peprotech, http://www.peprotech.com) for 24 h and spin-infected with concentrated virus in the presence of cytokines and polybrene (4 μg/ml) (Sigma, http://www.sigmaaldrich.com) [32]. Cells (1 × 10^6^) were transplanted intravenously into lethally irradiated (7.5 Gy) recipient mice, which were maintained on acidified water throughout the experimental period [32]. In some experiments, lin^−^c-kit^+^ hematopoietic progenitor cells sorted from C57BL6 mice were used, in place of 5-FU-treated BM cells. Results obtained using the two different sources of cells were essentially identical. For competitive repopulation, BM cells from the 5-FU-treated mice were infected with retroviruses for either GFP only, *AML1a,* or human CD4. Cells were then admixed to obtain mixtures of GFP-transduced and CD4-transduced cells, or AML1a-transduced and CD4-transduced cells for transplantation.

### Flow Cytometry of Mouse Cells

After red blood cell lysis, cells were incubated with the indicated antibodies, conjugated with either one of phycoerythrin, biotin, or allophyocyanin (APC); biotinylated antibodies were then stained with streptavidin-APC or streptavidin-peridinin-chlorophyll-protein Cy5.5. Nonspecific binding was blocked by preincubation with an anti-Fc receptor antibody (2.4G2). Antibodies used were as follows: anti-CD3 (145–2C11), anti-CD4 (RM4–5), anti-CD8 (53–6.7), anti-B220 (RA3-6B2), anti-CD19 (1D3 and MB19–1), anti-TER-119 (TER-119), anti-Gr-1 (RB6-8C5), anti-Mac-1 (M1/70), anti-c-kit (2B8), anti-CD34 (RAM34), anti-Sca-1 (D7), and anti-human CD4 (RPA-4). Isotype-matched Ig was used as a control. Antibodies were obtained from BD Biosciences (http://www.bdbiosciences.com) and eBioscience (http://www.ebioscience.com). Cells were washed with PBS containing 1% fetal calf serum and analysed on a FACSCalibur (BD Biosciences) with CELLQUEST software. Cell sorting was conducted with a FACSVantage (BD Biosciences).

### Western Blot Analysis

Total cell lysates were fractionated by SDS-PAGE and blotted onto a nylon membrane. AML1a and AML1b were detected with an anti-AML1 antibody (Ab-2, Oncogene Research Products, http://www.merckbiosciences.co.uk) or an anti-FLAG antibody (M2, Sigma). Anti-tubulin antibody (Sigma) was used to allow comparison of loaded protein amounts. Primary staining was visualized with a goat anti-rabbit Ig- horseradish peroxidase (HRP) conjugate, a goat anti-mouse Ig–HRP conjugate, or a secondary antibody by using enhanced chemiluminescence (Amersham Bioscience, http://www.amersham.com). Western blot analysis of primary human cord blood was conducted using a 12% acrylamide gel, and the AML1 proteins were detected using an anti-AML1 antibody (Ab-2) in combination with the highly sensitive West Femt reagent (Pierce, http://www.piercenet.com).

### In Vitro Colony-Forming Assays of Mouse Cells

Cells were plated in triplicate in methylcellulose under multimyeloid differentiation conditions (SCF [50 ng/ml], IL3 [10 ng/ml], IL6 [10 ng/ml], and erythropoietin [Epo, 3 units/ml]) (Methocult M33342, Stem Cell Technology, http://www.stemcell.com), or myelomonocytic differentiating conditions (SCF [50 ng/ml], IL3 [10 ng/ml], IL6 [10 ng/ml], and granulocyte/macrophage-CSF [GM-CSF; 10 ng/ml]). Colonies were typed and counted at day 7. For colony-forming unit (CFU) megakaryocyte (meg) (CFU-meg), α-MEM supplemented with 0.8% methylcellulose, 1% BSA, 30% FBS, 0.1 mM 2-mercaptoethanol, and 10 units of mouse thrombopoietin (Peprotech) was used [7].

### In Vitro Culture of Mouse Cells

Transduced cells were maintained in Iscove's modified Dulbecco's medium (Gibco, http://www.invitrogen.com) supplemented with 10% fetal calf serum in the presence of SCF (50 ng/ml) and IL3 (10 ng/ml). Cytokines were purchased from Peprotech.

### Cell-Cycle Analysis

Cells in culture were incubated with 10 μM of 5-bromo-2′-deoxyuridine (BrdU) (WAKO, http://www.wako-chem.co.jp/english) for 30 min and then fixed in 70% cold ethanol and treated with 4 N HCl. Incorporated BrdU was visualized by anti-BrdU antibody conjugated to fluorescein (BU.1, Chemicon International, http://www.chemicon.com) and analysed on a FACSCalibur along with DNA content as stained by propidium iodide.

### Long-Term Culture–Initiating Cell and Cobblestone Area-Forming Cells Assays for Mouse Cells

For long-term culture-initiating cell (LTC-IC) assays, GFP^+^ and GFP^−^ cells from the lin^−^ compartment were sorted from mouse BM by flow cytometry and inoculated onto pre-established irradiated stromal cells by limiting dilution in Myelocult medium (Stem Cell Technology). Cells were cultured for 4 wk and then subjected to colony formation assay in Methocult M33342, following the manufacturer's instructions. For cobblestone area-forming cell (CAFC) assays, the stromal cell line FBMD-1 (a kind gift from Dr. Gary Van Zant, University of Kentucky, http://www.uky.edu) was used. Total BM cells sorted on the basis of GFP expression were inoculated onto the pre-established stromal cell layer by limiting dilution and cultured for 5 wk.

### Cord Blood

Cord blood cells were collected, with written informed consent, for use in research. Total CD34^+^ cells and total CD15^+^ cells were purified using MACS magnetic beads (Miltenyi Biotec, http://www.miltenyibiotec.com) according to the manufacturer's instructions. CD34^+^ subsets were isolated by cell sorting using antibodies against human CD14, CD19, CD33, and CD133. For the isolation of CD36^+^ cells, purified CD34^+^ cells were cultivated in vitro with SCF (50 ng/ml), IL3 (20 ng/ml), and IL6 (20 ng/ml) for 7 d and isolated using a CD36-PE antibody in combination with anti-PE magnetic beads [33]. The resulting cells gave 98% erythroid output when plated in methylcellulose containing SCF, IL3, IL6, GM-CSF, G-CSF, and Epo.

### Quantification of AML1 Isoforms in Human Cord Blood by Reverse Transcription-PCR

Primers 5′-GAGGGAAAAGCTTCACTCTGA-3′ (primer C), GTGTACCGGGATCCATGCTA-3′ (primer 3A), and 5′-GTTGAGAGTCGACTGGAAAG-3′ (primer 2B) were used to detect *AML1a* (primers C and 3A) and *AML1b/AML1c* (primers C and 2B) as described [25]. PCR products thus obtained were then used as templates for additional PCR with the following primer sets to generate competitor DNAs: 5′-TTTGAATTCGAGGGAAAAGCTTCACTCTGAGAACCTCGAAGACATCGGCA-3′ (primer C′), 5′-TTTGAATTCGTGTACCGGGATCCATGCTA-3′ (primer 3A′), and 5′-TTTGAATTCGTTGAGAGTCGACTGGAAAG-3′ (primer 2B′). These primer sets were designed to generate products 157 bp shorter than the authentic PCR products and to facilitate subcloning. PCR products with primers C′ and 3A′, or with primers C′ and 2B′, were digested with EcoRI, and inserted into the EcoRI site of the pBluescript vector (Stratagene, http://www.stratagene.com) to obtain pBS/competitor A and pBS/competitor B. NotI/XhoI fragment of pBS competitor A was then inserted into NotI/XhoI site of the pMXs vector, then the EcoRV/XhoI fragment was inserted into the EcoRV/XhoI site of the pBS/competitor B. The plasmid thus obtained (pBS/competitor A + B) contained competitors for *AML1a* and *AML1b* in back-to-back orientation, with 1,250 bp of intervening spacer. This pBS/competitor A + B was used for the competitive PCR for both *AML1a* and *AML1b/AML1*c to quantitate AML1 isoforms in a given cell population [34]. Total RNA was isolated from the fractionated cord blood cells using Trizol reagent (Invitrogen, http://www.invitrogen.com) and then reverse-transcribed using random primers and Superscript II enzyme according to the manufacturers' instructions. cDNA thus obtained was then mixed with known amounts of the competitor plasmid and PCR was conducted either with primer sets C and 3A, or with primer sets C and 2B. PCR products were then fractionated on a 2.5% agarose gel electrophoresis, and the PCR products from endogenous *AML1* and from the competitor plasmid were quantitated densitometrically. The amounts of the endogenous *AML1* isoforms were then estimated as described [34] and normalized on the basis of G6PD expression estimated by a quantitative real-time PCR [35].

### Preparation of Lentivirus and Cord Blood Cell Transduction

An *AML1a* cDNA was cloned into the pHR-SINCSGW lentivirus [36], which carries an emerald-GFP reporter. Lentiviruses were pseudotyped with the vesicular stomatitis virus G (VSVG) protein by transient transfection of 293T cells. High-titre viral stocks were prepared by ultracentrifugation [37]. CD34-enriched cord blood cells were infected for 24 h by lentivirus (either *AML1a* or control virus) with MOI of 100 in serum free medium Stem/Span (Stem Cell Technologies) supplemented with SCF (100 ng/ml), FLT-3 ligand (100 ng/ml), thrombopoietin (20 ng/ml), and IL6 (20 ng/ml) (all growth factors were from Peprotech) [38,39].

### In Vitro Analysis of Human Cells

Colony-forming cell (CFC) assays with human cells were under serum-free conditions with recombinant human SCF, GM-CSF, IL3, IL6, G-CSF, and Epo (MethoCult H4536, StemCell Technologies). LTC-IC assays with human cells were performed in 96-well format in Myelocult H5100 medium (StemCell Technologies) on a feeder layer comprising a 1:1 mixture of irradiated M210B4 and S1/S1 mouse fibroblasts [40]. For limiting dilution CAFC assays MS-5 stroma cells [41] were maintained in α-MEM medium complemented with 2 mM L-glutamine, 2 mM sodium pyruvate, 10% FBS (Stem Cell Technologies). After the cells were inoculated, weekly half medium changes were performed for the duration of the culture. CFAC were scored at 6 wk [42,43].

### Nonobese Diabetic–Severe Combined Immunodeficient Mice and Transplantations

Nonobese diabetic–severe combined immunodeficient (NOD-SCID) mice were bred and maintained at the Weatherall Institute of Molecular Medicine (WIMM) animal facility in accordance with Home Office regulations (United Kingdom). Animals were handled under sterile conditions. Transplantations were performed by tail-vein or intrafemoral injection into 6- to 10-wk-old mice. Recipients received 350 cGy of total body irradiation, and BM engraftment was assessed at 8 wk post-transplantation. Human cell engraftment was assessed by staining of BM cells with anti-CD19-APC (DAKO), anti-CD34-PE (BD Biosciences Pharmingen), anti-CD45-APC (DAKO, http://www.dako.com), and CD38-FITC (DAKO).

## Results

### Effects of *AML1a* and *AML1b* on Long-Term Engrafting Cells and Hematopoiesis In Vivo

**Figure 1:**
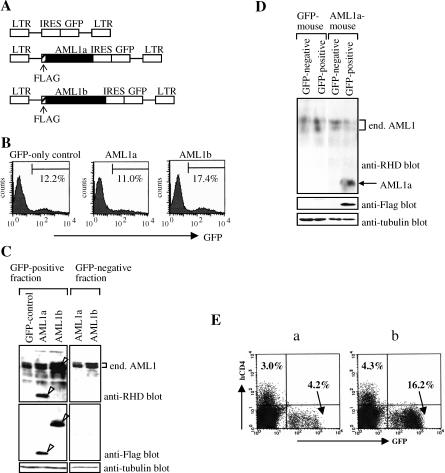
Transduction of Mouse HSCs with *AML1* Isoform-Containing Retroviruses (A) Recombinant viruses. (B) FACS analysis of GFP expression in infected hematopoietic cells immediately after infection with control, *AML1a*-containing, or *AML1b*-containing viruses. (C) Western blot analysis of lysates from cells sorted immediately after infection by using anti-AML1, anti-Flag, and anti-tubulin antibodies. The anti-AMLl antibody was raised against the Runt domain and therefore detected both human and endogenous mouse AML proteins. (D) Western blot analysis of GFP^+^ and GFP^−^ BM cells from animals 4 mo after the transplantation (antibodies as in [C]). (E) Typical FACS analyses of BM cells from competitive repopulation assays of *AML1a*-transduced cells. BM cells of mice transplanted with mixtures of human CD4^−^ and GFP-only-control-transduced cells (a) or human CD4^−^ and *AML1a*-transduced cells (b) were analysed for expression of human CD4 and GFP.

BM cells were isolated from 5-FU-treated mice and transduced with murine stem cell virus -based retroviruses containing amino-terminal FLAG-tagged *AML1a* or *AML1b* inserted upstream of an IRES-GFP cassette (Figure 1A). GFP^+^ and GFP^−^ cells (Figure 1B) were separated by flow cytometry, and expression of AML1a and AML1b were confirmed by Western blotting using an antibody directed against a peptide within the AML1 runt domain (Figure 1C), which also detects endogenous murine Aml1; the identity of the AML1a and AML1b were additionally confirmed by Western blotting with an antibody directed against the FLAG tag (Figure 1C). These results also showed that the expression level of exogenous AML1a and AML1b was less than 2-fold that of endogenous Aml1 in the cells used subsequently for transplantation.

Animals were transplanted in cohorts to facilitate kinetic studies of engraftment (Table 1). The contribution of control vector-transduced cells was relatively stable over time in all cohorts followed. *AML1b*-transduced cells, in contrast, never contributed to BM at 1 mo or thereafter. Strikingly, the contribution of *AML1a*-transduced cells increased with time; in a typical case (cohort 4), percentage GFP was 10.5% at 3.5 mo, 11.4% at 4 mo, 42.2% at 11.5 mo, and 53.4% at 12 mo. Western blotting showed that exogenous AML1a expression was 2- to 3-fold higher than endogenous Aml1 and confined exclusively to GFP^+^ cells within the graft (Figure 1D). These results suggest that while AML1b expression abrogates engraftment potential, AML1a expression confers a growth advantage on BM cells in vivo.

**Table 1:**
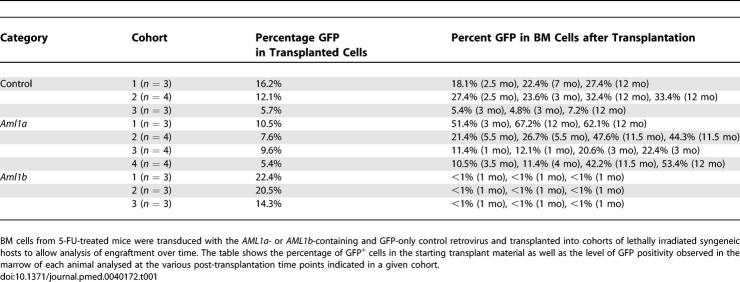
Kinetic Analysis of Engraftment of *AML1a*-, *AML1b*-, and Control Vector-Transduced BM Cells

We therefore examined this growth advantage using competitive repopulation assays. Competitor cells marked by expression of the extracellular domain of a human CD4 were mixed with either *AML1a*- or control vector-transduced cells just prior to transplantation into cohorts of lethally irradiated mice. In one cohort, a mixture of 6.2% of GFP^+^ control vector-transduced cells and 4.0% of human CD4^+^-transduced competitor cells were used for the transplantation. In the second cohort, a mixture of 4.5% GFP^+^ *AML1a*-transduced cells and 5.7% of human CD4^+^-transduced competitor cells were used. Subsequent analysis of engrafted cells—a typical example is shown in Figure 1E—revealed the engraftment potential of AML1a-expressing cells to be substantially higher than their control-vector transduced counterparts. Kinetic studies conducted over a 9-mo period (Table 2) showed the ratio of GFP and CD4 to be relatively stable in the control-vector/CD4 cohort. In contrast, the GFP/CD4 ratio increased with time in the AML1a/CD4 mice cohort (Table 2). This property was also evident in the secondary transplantation experiments: when BM cells from a primary recipient exhibiting GFP/CD4 ratio = 1.6 at 3 mo post-transplant were secondarily transplanted, the secondary recipients displayed ratios of 3.0 ± 1.41 (mean ± standard deviation) at 3 mo and 9.0 ± 3.29 at 8 mo post-transplant. This increase in the GFP/CD4 ratio was not seen in mice secondarily transplanted with BM cells taken from control (control-vector/CD4) cohorts. Collectively, these results suggest that expression of AML1a confers a high level of competitive engraftment potential.

**Table 2:**
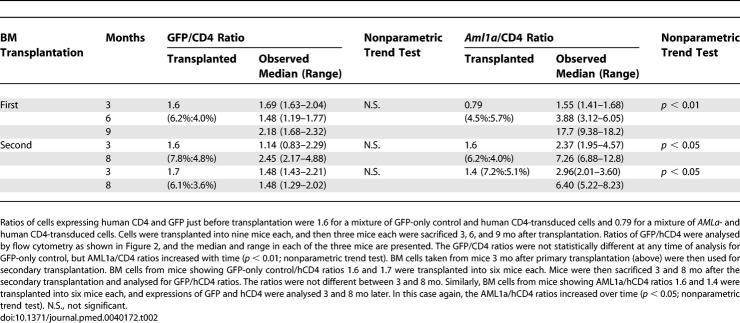
Competitive Repopulation Assays of *AML1a*-Transduced Cells in First and Second Transplantations

**Figure 2:**
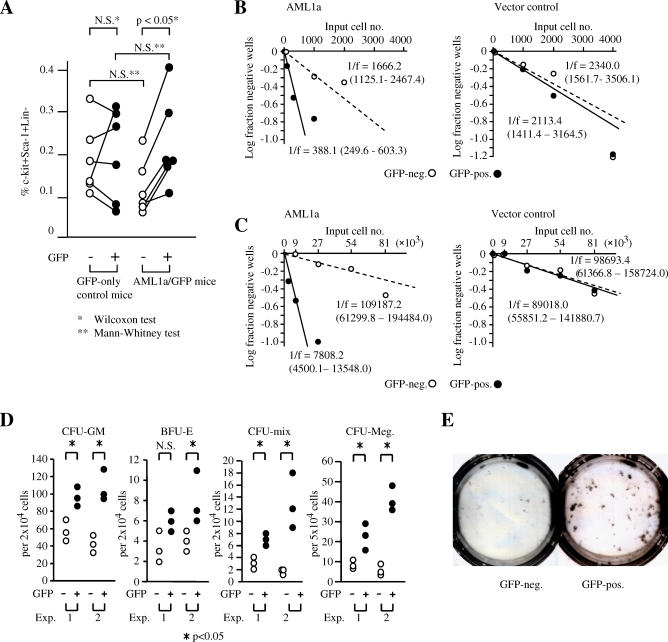
Analysis of BM Compartments in Engrafted Animals Results from GFP^+^ and GFP^−^ cells are shown in solid and open symbols, respectively. (A) Percentages of KSL cells in GFP^+^ and GFP^−^ fraction of individual animals are presented pairwise. All animals were analysed 4 mo after transplantation. Statistical significance was evaluated using Wilcoxon and Mann-Whitney tests. (B) LTC-IC analysis of GFP^+^ and GFP^−^ cells from the lin^−^ BM compartment of *AML1a* and control animals. LTC-IC frequency within the lin^−^ compartment and its 95% confidence interval (in parenthesis) are presented. Two independent experiments gave a similar result. (C) CAFC analysis of GFP^+^ and GFP^−^ cells from total BM of *AML1a* and control animals. Wells containing clusters of six or more cobblestone cells after 35 d of culture were scored as positive, and the frequency of CAFC calculated by Poisson statistics; CAFC frequency and its 95% confidence interval (in parenthesis) are presented. Two independent experiments gave a similar result. (D) Colony-forming activity BM cells sorted from an *AML1a* animal and cultured under multimyeloid (CFU-GM, erythropoietic burst formation [BFU-E], and CFU-mix) and megakaryocytic (CFU-meg) conditions are shown. Representative data from three independent experiments conducted in triplicate are presented. Asterisks indicate statistical significance of the difference estimated by Mann-Whitney test. (E) Photomicrographs show relative sizes of colonies generated in multimyeloid condition.

We explored this competitive potential further by examining the status of the primitive progenitor compartment in primary *AML1a* recipients. We determined the number of c-Kit^+^Sca-1^+^Lin^−^ (KSL) cells within GFP^+^ and GFP^−^ fractions of BM in each of six *AML1a* and six control animals 6 mo post-transplantation, and the results are shown pairwise for individual mice in Figure 2A. No difference in KSL frequency was observed between GFP^+^ and GFP^−^ fractions from control animals. In contrast, the GFP^+^ fractions of *AML1a* mice contained significantly more KSL than the corresponding GFP^−^ fractions of the same animals. Interestingly, however, percentage KSL was not significantly different between the GFP^+^ fractions of *AML1a* and control animals. This result may reflect the relative competitiveness of GFP^+^ and GFP^−^ cells within the BM of a single animal, or alternatively percentage KSL may not entirely reflect the frequency of functional stem and multipotent progenitors. We therefore quantitated the number of functional primitive hematopoietic cells using LTC-IC and CAFC assays, and typical data from one such experiment are shown in Figure 2B and 2C. In the case of LTC-IC assays, GFP^+^lin^−^ and GFP^−^lin^−^ cells were isolated from transplanted animals by flow cytometry and cocultured using the limiting dilution method on pre-established BALB/c mouse BM stromal layers. After 4 wk of culture, the cells were harvested and assayed for CFC activity to enumerate the frequency of primitive hematopoietic cells. In the case of CAFC assays, total GFP^+^ or GFP^−^ cells were purified from transplanted mice and cultured using the limiting dilution method on FBMD-1 cell line stromal layers; cobblestone areas, comprising six or more cells, were enumerated 5 wk later. These in vitro assays revealed a significant increase in the frequency of primitive hematopoietic cells in AML1a-positive BM-fraction; comparison of the GFP^+^ versus GFP^−^ fractions showed a 4-fold increase (1/388.1 versus 1/1,666.2) in LTC-IC (Figure 2B) within lin^−^ cells and a 14-fold increase (1/7,808.2 versus 1/10,9187.2) in CAFC (Figure 2C) within unfractionated cells. These increases were not seen in the corresponding fractions of cells from control animals (Figure 2B and 2C).

We next examined the committed progenitor compartment using CFC assays. GFP^+^ cells from *AML1a* mice displayed increased total CFC activity (Figure 2D) primarily in granulocyte- and/or macrophage-restricted (CFU-GM) and primitive mixed lineage (CFU-mix) progenitors. Furthermore, AML1a-expressing colonies (GFP^+^) were larger than those produced from control cells (GFP^−^) from the same animals (Figure 2E). AML1a expression did not alter the frequency of erythroid colonies (erythropoietic burst formation) generated but was associated with an increase in CFU-meg formation (Figure 2D), but cells in these CFU-meg colonies were smaller than controls, displaying hypolobulated nuclei indicative of impaired differentiation (unpublished data). Consistent with these CFC data, the lin^−^c-kit^+^ progenitor compartment was increased 4.0-fold in the GFP^+^ versus GFP^−^ fraction in animals transplanted with *AML1a*-transduced BM, and these animals also displayed increases in immature blast-like cells (Figure S1). Morphological examination of sorted BM cells from *AML1a* and control mice also revealed an over-representation of myeloid versus lymphoid cells in the GFP^+^ fraction of *AML1a*-mice (Figure S1A), which was confirmed by immunophenotyping (unpublished data). There were no morphological or immunophenotypic indications of arrested differentiation in the *AML1a*-expressing myeloid compartment (Figure S1B and S1C).

### *AML1a* and *AML1b* Display Opposing Effects on Myeloid Differentiation In Vitro

**Figure 3:**
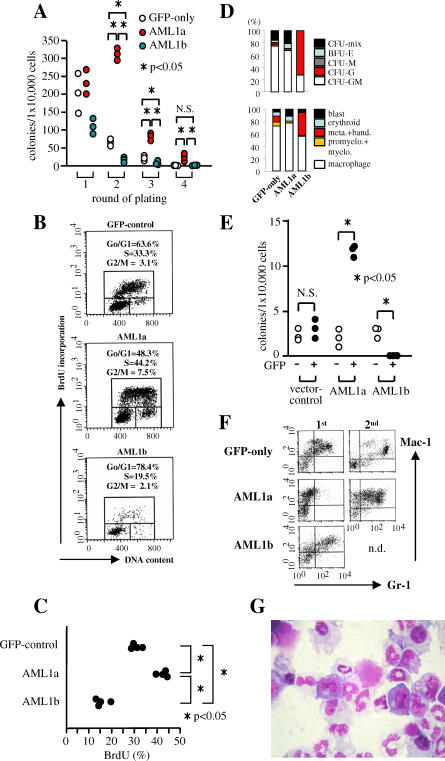
Myeloid Potential of *AML1a*- or *AML1b*-Transduced Cells (A) Frequency of CFCs in serial replating experiments; we sorted 1 × 10^4^ GFP^+^ cells immediately after infection with GFP vector-only, *AML1a*-containing, or *AML1b*-containing viruses, and they were plated. For replating, cultures were harvested on day 7 and 1 × 10^4^ cells replated. Representative data from no fewer than three independent experiments conducted in triplicate are presented. Kruskal-Wallis test showed statistical significance of difference (*p* < 0.05) of colony numbers generated by the three populations (GFP, *AML1a,* and *AML1b*) in rounds 2, 3, and 4, but not in round 1. Difference of any given two populations was therefore analysed in rounds 2, 3, and 4, by Mann-Whitney test, with asterisks indicating statistical significance of the difference. (B) Representative cell-cycle analyses of control and *AML1a*- or *AML1b*-transduced cells from the second round of replating are presented. Cells harvested from CFC assays were pulsed with BrdU and analysed for BrdU incorporation along with DNA content. (C) Graphical summary of cell-cycle data from four independent experiments. Kruskal-Wallis test showed statistical significance of difference (*p* < 0.01) of the three populations (GFP, *AMl1a,* and *AML1b*) and Mann-Whitney tests showed statistical significance of the difference between any given two populations (*p* < 0.05). (D) Colony types formed in multimyeloid conditions (upper bar graph) are shown. After typing on day 7, cultures were harvested for cytospin preparations and differential counting (lower bar graph). (E) CFU-meg formation was analysed. BM cells from 5-FU-treated BALB/c mice were infected with viruses for vector-alone, *AML1a,* and *AML1b,* and then sorted into GFP^+^ and GFP^−^ cells for colony assays (triplicate). Two experiments yielded a similar result. Asterisks indicate statistical significance of the difference estimated by Mann-Whitney test. (F) FACS analysis for myeloid-differentiation markers (Gr-1 and Mac-1) on cells harvested from colonies at the first and second replating. (G) Photomicrograph of *AML1a*-transduced cells harvested from second replating.

The abrogation of engraftment by *AMLb* precluded further analysis of its effects on haematopoietic cells in vivo. We therefore compared effects of *AMLb* and *AML1a* on the clonogenic activity of appropriately transduced 5-FU cells (see Figure 1A and 1B) in vitro using CFU in culture assays. The total number of colonies was similar in *AML1a*- and control vector-transduced cells, but significantly reduced in AML1b-expressing cells (Figure 3A). Upon serial replating—an assay of self-renewal—AML1b-expressing cells gave rise to significantly fewer colonies than control vector-transduced cells at all passages examined, whereas expression of AML1a produced more colonies. Furthermore, compared with colonies produced by control vector-transduced cells, colonies from AML1a-expressing cells were larger, whereas colonies from AML1b-expressing cells were smaller (Figure S2A). Analysis of cell-cycle status, as assessed by using BrdU incorporation and DNA content, revealed that AML1a-expressing cells were more proliferative than controls or AML1b-expressing cells (Figure 3B and 3C). Results of colony typing and morphological assessment of cells within primary colonies are shown in Figure 3D. AML1b-expressing cells produced no erythroid output and gave rise to significantly more granulocyte-restricted CFU (CFU-G) than control cells. The overall distribution of colony type produced by AML1a-expressing cells was similar to that seen in controls, but the granulocytic component within CFU-GM colonies was markedly decreased (unpublished data). AML1b-expressing cells did not produce CFU-meg colonies (Figure 3E). In contrast, AML1a cells produced more CFU-meg than controls (Figure 3E). When compared with vector-transduced control cells, the Gr-1-high mature granulocytic fraction was increased by AML1b expression and decreased by AML1a expression in both multimyeloid and myelomonocytic conditions. However, upon replating, AML1a-expressing cells started to express high levels of Gr-1 (Figure 3F), which was accompanied by the appearance of morphologically mature granulocytes (Figure 3G). Similar results were obtained when cells were plated under myelomonocytic- (SCF + IL3 + IL6 + GM-CSF) promoting culture conditions (unpublished data).

**Figure 4:**
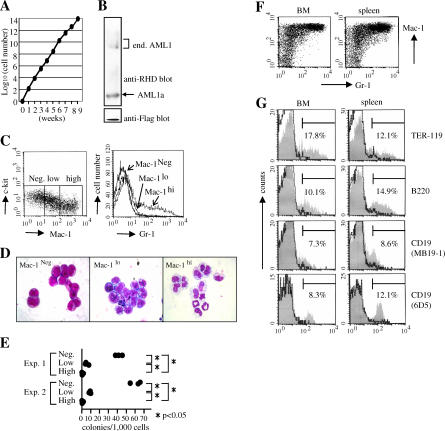
Extended Culture and Engraftment Potential of *AML1a*-Transduced Cells Cells harvested from the third replating of *AML1a*-transduced cells were cultured in medium containing SCF + IL3. Cells were counted weekly and replated at 1 × 10^5^ cells per millilitre in fresh medium and cytokines. Successful long-term cultures were established in four out of four experiments using either cells derived from BALB/c or C57BL6 mice. (A) Fold increase of cell number are presented on a logarithmic scale versus time. (B) Western blot analysis of cultured *AML1a*-transduced cells using anti-AML1 (anti-RHD) and anti-FLAG antibodies. (C) Flow cytometric analyses of the cultured *AML1a*-transduced cells using anti-c-kit and anti-Mac-1 (left) and anti-Gr-1 and anti- Mac-1 (right) antibodies. (D) May-Grunwaldt-Giemsa staining of the sorted Mac-1^−^, Mac-1^low^, and Mac-1^high^ cells in culture. (E) CFC activity of sorted Mac-1^−^, Mac-1^low^, and Mac-1^high^ cells in SCF and IL3. Typical data from three independent experiments conducted in triplicate are presented. Kruskal-Wallis test showed statistical significance (*p* < 0.05) of the three populations (Mac-1^−^, Mac-1^low^, and Mac-1^high^), and Mann-Whitney tests showed statistical significance of the difference (*p* < 0.05) of any given two populations. (F and G) Engraftment and lineage potential were analysed. Mice were lethally irradiated and transplanted with 10^7^ cultured AML1a-transduced cells and 5 × 10^5^ fresh “radioprotective” BM cells to mitigate against immediate effects of lethal radiation. BM and spleen cells were gated for GFP and analysed for lineage markers ten days after transplantation. Gr-1 (granulocytic) and Mac-1 (myeloid) expression in BM and spleen were analysed (F). Given the predominance of Mac-1^+^ cells in the graft, Mac-1^−^ cells were analysed for TER-119 (erythroid), B220 (B-lymphoid), and CD19 (B-lymphoid) (G). Grey shading shows marker expression, and the black dotted lines show profiles of isotype-matched control antibodies. Representative data from three independent experiments are shown.

Collectively, these findings suggest that AML1b expression promotes granulocytic differentiation at the expense of alternative lineages, and AML1a expression retards but does not block granulocytic maturation of BM cells under the conditions used while promoting self-renewal and proliferation of progenitor cells. Furthermore, *AML1a*-transduced cells recovered from the third round of replating propagated in suspension culture, displaying a cumulative cell expansion of about 10^14^-fold in 9 wk (Figure 4A). The cultures comprised an adherent layer of macrophages and a mixture of floating monocytic, granulocytic, and blast-like cells (Figure S2B). These cultures were reproducibly established in both BALB/c and C57BL6-derived cells and continued to express exogenous AML1a as evidenced by Western blotting with anti-RHD and anti-FLAG antibodies (Figure 4B). Flow cytometric analysis of the nonadherent cells revealed the following subpopulations; (1) Mac-1^−^/c-kit^+^, (2) Mac-1^low^/c-kit^low^, (3) Mac-1^high^/c-kit^−^/Gr-1^−^, and (4) Mac-1^high^/c-kit^−^/Gr-1^+^ (Figure 4C). Expression of lymphoid (CD3, CD4, CD8, CD5, CD25, B220, CD19, IL7-receptor alpha, AA4.1, BP-1, and NK1.1) and erythroid (TER-119) markers was not detected; a proportion of Mac-1^−^/c-kit^+^ cells expressed Sca1 (unpublished data). Morphological analysis (Figure 4D) and colony-forming assays (Figure 4E) showed Mac-1^−^ cells to be blast-like with a colony-forming efficiency of 4%–7%, Mac-1^low^ cells to be immature myeloid cells with a lower clonogenic capacity (0.5%–0.7%), and Mac-1^high^ cells to be a mixture of monocytic and granulocytic cells with no colony-forming potential. The Mac-1^high^/Gr-1^+^ fraction contained exclusively granulocytes as judged by morphology and enzymatic assays (unpublished data). Finally, fluorescence-activated cell sorter (FACS)-purified Mac-1^−^/c-kit^+^ cells were able to generate all of the other cell types observed in the cultures (unpublished data). The use of Epo or ST-2 stromal cells to obtain in vitro erythroid or lymphoid outputs, respectively, was not successful, however (unpublished data). These results suggest that AML1a-expressing Mac-1^−^/c-kit^+^ cells are capable of self-renewal and myelomonocytic differentiation in vitro.

We next transplanted cells from these proliferating in vitro cultures into lethally irradiated syngeneic mice together with a radioprotective dose of fresh BM cells. We found 30%–40% of harvested spleen and BM cells expressed GFP, demonstrating successful engraftment of cultured AML1a-transduced cells 10 to 20 d after the transplantation (unpublished data). Engraftment was predominantly myelomonocytic (Figure 4F), but GFP^+^ cells contributed to the erythroid (TER-119) and B-lymphoid (B220, CD19) lineages; given the predominance of myelomonocytic Mac^+^ cells within the GFP^+^ fraction, this contribution to erythroid and B-lymphoid lineages is most clearly apparent in the GFP^+^/Mac-1^−^ fraction (Figure 4G). GFP^+^ cells did not contribute, however, to the thymus, nor were CD3^+^ cells seen in BM or spleen. These findings demonstrate that the cultured *AML1a*-transduced cells have erythroid and B-lymphoid as well as myeloid differentiation potential. The engraftment of these cultured *AML1a*-transduced cells was not persistent, however, with virtually no GFP^+^ cells being detectable in the BM or spleen of the transplanted animals at 5 wk post-transplant and thereafter. Collectively, these data suggest that AML1a expression maintains short-term reconstituting multipotent progenitors prolonged in vitro culture.

### Analysis of AML1 Isoforms in Human Cord Blood

**Figure 5:**
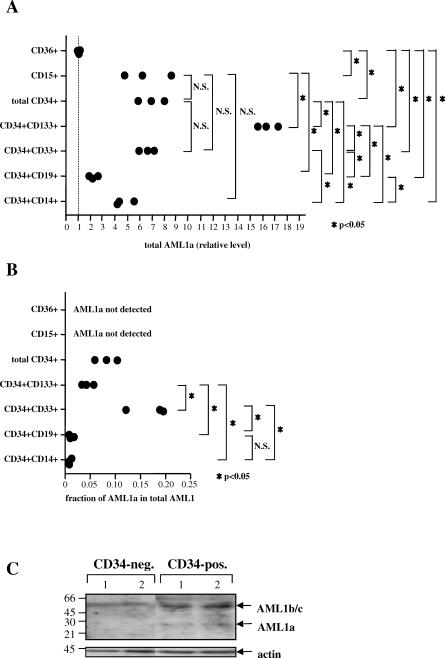
Expression of AML1 Isoforms in Human Hematopoietic Compartments Human blood cells from three pooled individual umbilical cord samples were sorted on the basis of the cell surface molecules indicated, and the levels of transcripts corresponding to different AML1 isoforms were quantitated in triplicate and normalized relative to G6PD. (A) Total *AML1* transcript levels are presented as relative levels of that observed in CD36^+^ cells. (B) The fraction of *AML1a* relative to total *AML1* transcripts was determined. In (A) and (B) Kruskal-Wallis test showed statistical significance of difference among the fractions (*p* < 0.01). Mann-Whitney tests were then used to estimate the difference of any given two fractions, with asterisks indicating the statistical significance (*p* < 0.05). N.S., not significant. (C) Western blot analysis of AML-isoform expression using the anti-RHD antibody in CD34^+^ and CD34^−^ fractions of human cord blood (4 × 10^5^ cells each) from two individual healthy donors. Analysis of actin expression in the same samples provide a control for loading.

**Figure 6:**
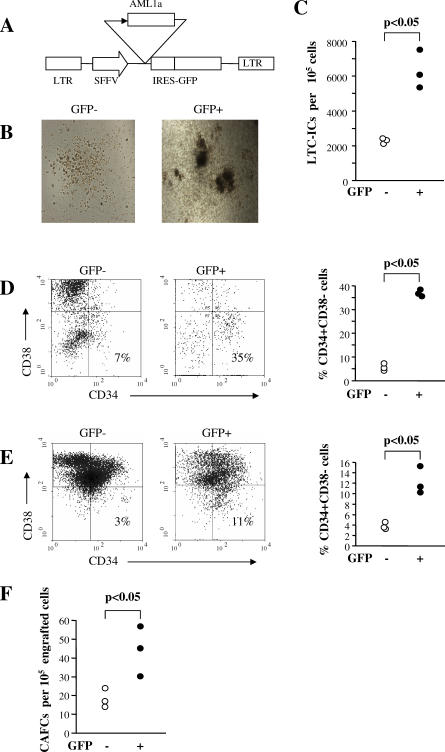
Forced Expression of *AML1a* in Human Cord Blood Cells (A) Lentiviral construct used for transduction. (B) Photomicrograph of typical LTC-IC colonies derived from AML1a-expressing (GFP^+^) and control (GFP^−^) human cord blood cells. (C) Frequency of LTC-IC in AML1a-expressing (GFP^+^) and control (GFP^−^) human cord blood cells. (D) Representative FACS analysis of *AML1a*-transduced (GFP^+^) and control (GFP^−^) cells recovered after 5 wk of culture on MS-5 stromal layers. Right plot shows graphical summary of all experiments performed. (E) Representative FACS analysis of *AML1a*-expressing (GFP^+^) and control (GFP^−^) human cells engrafted in the BM of NOD-SCID mice at 8 wk (left plot). Right plot shows graphical summary of all experiments performed. (F) Frequency of LTC-IC in human cells derived from primary NOD-SCID mice engrafted with *AML1a*-transduced cells. In (C), (D), (E), and (F), Mann-Whitney test showed statistical significance of the difference (*p* < 0.05).

Taken together, the data obtained in mouse cells raised the possibility that AML1 may have some utility in the setting of expansion of human cord blood-derived stem and progenitor cells. We therefore next assessed the expression of AML1 isoforms in different cellular compartments of human cord blood, which is a rich source of stem and progenitor cells. For these experiments a quantitative competitive reverse transcription-PCR strategy was used (see Methods). Total AML1 (AML1a + AML1b/c) expression was similar in progenitor (CD34^+^) and myelomonocytic (CD15^+^) subsets, and by comparison substantially was reduced in the erythroid (CD36^+^) compartment (Figure 5A). In contrast, AML1a expression was only observed in progenitor (CD34^+^) but not myelomonocytic (CD15^+^) or erythroid (CD36^+^) compartments (Figure 5B). CD34^+^ cells were therefore subfractionated into CD133^+^ (primitive), CD33^+^ (immature), CD19^+^ (B-lymphoid), and CD14^+^ (macrophage/monocyte) progenitor subsets. Total AML1 expression was highest in CD34^+^CD133^+^ cells, followed by CD34^+^CD33^+^, then CD34^+^CD14^+^, and finally CD34^+^CD19^+^ cells (Figure 5A). The AML1a to AML1b/AML1c ratio was about 1:6 in CD34^+^CD33^+^, 1:30 in CD34^+^CD133^+^, and 1:100 in both CD34^+^CD19^+^ and CD34^+^CD14^+^ cells (Figure 5B). Collectively, these findings suggest that *AML1* is relatively abundant in stem/early myeloid progenitors and under-represented in erythroid progenitors. Thus, while expression of *AML1b/AML1c* predominated in all fractions analysed, *AML1a* expression was restricted to CD34^+^ progenitor compartments and within these were relatively most enriched in the CD34^+^CD33^+^ subset. We next examined expression of AML1 isoforms in cord blood cells at the level of protein. Cell extracts of CD34^+^ and CD34^−^ CB cells from two different individuals were analysed by Western blotting. Although technically difficult given the low cells numbers available, the results suggest that *AML1a* expression is restricted to the CD34^+^ compartment where it constitutes a significant fraction of the total AML expressed (Figure 5C). These expression data, particularly the protein data, raise the possibility that expression of AML1a may be relevant to the function of primitive human haematopoietic cell compartments. We explored this possibility functionally by constructing a lentiviral expression vector for *AML1a* (Figure 6A), using it to transduce CB cells, and subsequently assessed their behaviour in vitro and in vivo. *AML1a* lentivirus-transduced CD34^+^CD38^−^ human CB cells were initially assessed in LTC-IC assays on M2-10B4 and SI/SI stromal layers. AML1a-expressing (GFP^+^) colonies appeared larger than their GFP-control counterparts (Figure 6B), and LTC-IC frequency was significantly enriched (approximately 3-fold) in the GFP^+^ fraction (Figure 6C). Similar results were obtained in CAFC assays conducted on MS-5 stromal cells (unpublished data), and in this case cells could be retrieved from the culture and examined immunophenotypically. The results show an increase (approximately 9-fold) in the proportion of primitive cells (CD34^+^CD38^−^) within the GFP^+^ fraction (Figure 6D). In light of these observations, we assessed the functional potential of *AML1a*-transduced cells in a xenotransplant setting. CD34^+^CD38^−^ CB cells were transduced with AML1a lentivirus and injected into irradiated NOD-SCID recipients, and human cell engraftment assessed at eight weeks. AML1a-expressing cells successfully engrafted, and more importantly, the proportion of CD34^+^CD38^−^ cells was approximately 3-fold higher within the GFP^+^ fraction of the graft (Figure 6E). Consistent with this, functional assessment of primitive cell activity within the GFP^+^ and GFP^−^ fractions of the graft using LTC-IC assays revealed a 3-fold increase in the GFP^+^ (i.e., AML1a-expressing) population (Figure 6F). Taken together, these results indicate that *AML1a* has the capacity to enhance primitive hematopoietic cell function in human cord blood both in vitro and in vivo.

## Discussion

Here, we show that *AML1a* increases the competitive engraftment potential and in vitro proliferation capacity of primitive BM compartments, while *AML1b* exhibits an essentially opposing activity, promoting differentiation and abrogating engraftment. Expression of *AML1a* in human cord blood-derived hematopoietic cells gave results broadly in line with those obtained in mouse cells.

Although *AML1/RUNX1* has been shown to be critically involved in the generation and maintenance of hematopoietic stem and progenitor cells as well as the differentiation of their progeny, the precise roles of its different isoforms in these processes is not as yet fully understood [44,45]. Most studies have focused on the role of *Aml1* in the specification of HSC either in knockout mice or using the hematopoietic differentiation of embryonic stem cells as a model [46–48]. These studies indicate that expression of Aml1b or an Aml1b molecule that lack its carboxyl-terminal (VPRWY) repression domain is sufficient to rescue definitive hematopoiesis in *Aml1*-deficient cells or animals. In contrast AML1 moieties that lack the transactivation domain, and hence may be considered AML1a-like, are unable to rescue hematopoiesis. Elegant as these studies are they do not directly address the physiological role of *AML1a* in homeostatic adult hematopoiesis, its potential as a nonphysiological but therapeutic regulator of the adult stem and progenitor compartment, nor its pathological activity in leukaemia. Here we have approached these issues and provided evidence that the AML1a and AML1b isoforms have opposing effects on hematopoietic stem and progenitor cells. Most notably, in murine cells we have shown that expression of *AML1b* abrogates hematopoietic reconstitution in an experimental transplant setting, whereas expression of AML1a significantly enhances competitive engraftment potential in both primary and secondary recipients. In mice transplanted with *AML1a*-transduced cells, the frequency of primitive progenitors was significantly enlarged. CAFC activity was increased 14-fold relative to controls, and the frequency of LTC-IC in the lin^−^ compartment increased 4-fold; since the lin^−^ compartment was itself more than 3-fold increased, the overall increase in total LTC-IC was at least 12-fold in *AML1a*-transduced versus untransduced cells. Committed progenitor activity (as judged by CFC assays) in the *AML1a*-expressing component of the grafts was also increased, but to a lesser degree, and the colonies produced were larger, suggesting increased proliferation, but they exhibited no block in myeloid differentiation. Since AML1b abrogated engraftment, further comparison with AML1a was necessarily conducted in vitro. Here AML1b promoted granulocytic differentiation whereas AML1a enhanced self-renewal as judged by colony replating and gave rise to larger colonies, which were somewhat retarded, although not arrested, in granulocyte differentiation.

Cell-cycle analysis suggested that this growth advantage was the result of increased proliferation as opposed to decreased apoptosis. These in vitro effects of AML1a are broadly in agreement with results obtained by forced expression of AML1a in myeloid cell lines in which granulocytic differentiation was blocked, and this blockade was accompanied by uncompromised cell growth [26]. Most remarkably, *AML1a*-transduced BM cells were able to expand in a simple culture with SCF and IL3, with immature (c-kit^+^lin^−^) cells proliferating vigorously yet retaining the ability to produce terminally differentiated myelomonocytic cells in vitro and giving rise to short-term multilineage engraftment in vivo. These extended in vitro cultures established by *AML1a*-transduced cells thus recapitulate many aspects of hematopoiesis, retaining multipotency in a manner reminiscent of human CD34^+^ cells programmed to express the AML1-ETO oncoprotein [49].

Both *AML1a* and *AML1-ETO* are thought to interfere with *AML1b/c,* and possibly also *AML2* and *AML3,* in a dominant-negative fashion, either by directly competing for AML-binding sites on DNA or by sequestering subunit b of core binding factor (CBFb), an obligate subunit required for binding of AML proteins to DNA [3]. Thus comparison of the results obtained with our *AML1a* mice to those reported for *AML1-ETO* and *Aml1*-targetted animals should be informative with respect to understanding physiological and pathological AML1 activities. Like *AML1a* animals, “haploinsufficient” *Aml1* ^+/−^ mice also exhibit increased CFU-GM and CFU-mix, but unlike *AML1a* mice, the size of colonies generated is unaffected [50]. BM cells of the *Aml1* ^+/−^ mice exhibit increased competitiveness accompanied by a nearly doubled frequency of immature hematopoietic stem and progenitors as assessed in CAFC assays [50]. The KSL fraction is increased in the BM cells of conditional *Aml1* ^−/−^ mice, and hematopoietic reconstitution ability in transplanted mice maintained; yet *Aml1* ^−/−^ hematopoietic cells are defective in competitive repopulation capacity [8]. Mice reconstituted with *AML1*a-transduced cells did not show appreciably inhibited myeloid differentiation, in contrast to mice reconstituted with hematopoietic cells retrovirally transduced with *AML1-ETO* [51,52]. These findings imply that while some aspects associated with forced AML1a expression mimic reduced dosage, although not complete loss, of Aml1, AML1a confers unique effects on stem and progenitor cell properties that cannot be achieved by reducing the dosage of the *Aml1* gene or by expression of the AML1-ETO oncoprotein. Of note here also are our preliminary results obtained with a mutant of *AML1 (AML1R139G),* which can sequester *CBFb* but cannot bind DNA [53]. Like *AML1a, AML1R139G*-transduced cells exhibited delayed granulocytic differentiation in vitro. Unlike *AML1a, AML1R139G*-transduced cells did not produce long-term cultures in vitro and did not contribute to long-term hematopoiesis in vivo (ST, unpublished data). Thus, the ability of AML1a to bind DNA appears crucial for cell expansion, thereby further emphasising that AML1a is not a simple antagonist of AML1b/c or AML2/AML3, but rather exerts distinct effects on hematopoietic cells by virtue of its DNA-binding activity. Although we have in this study concentrated on the role of AML1a, the observation that AML1b can abrogate engraftment, presumably through enhancing granulocytic differentiation, is of interest and perhaps at first glance surprising. However it is now well documented that transcription factor effects are highly dose sensitive. Aml1 is itself a case in point [54], and GATA-2 [55,56], PU1 [57], and OCT4 [58] provide additional examples. Recent modelling approaches [59] (S. Huang and TE, unpublished data) are starting to illuminate how dose-sensitive bi-stable switches can be created as a result of the transcriptional motifs (coherent and incoherent feedforward motifs, crossantagonistic motifs, and the like) adopted by transcription factors. While such dynamic models and the genetic regulatory networks on which they are based [60] do not as yet include *Aml1,* the fact that Aml1is a key regulator of granulocyte/monocyte-affiliated growth factor receptors as well as transcription factors provides a plausible framework for understanding how increasing its expression could lead to enhanced differentiation at the expense of self-renewal.

In light of the AML1a activities highlighted by our experiments, it is intriguing that *AML1a* transcripts are evident in selected samples of leukaemia [23]. *AML1* mutants devoid of the carboxyl-terminal portion of the molecule, and thereby structurally resembling *AML1a,* are also found in BM cells of some patients with myelodysplastic syndrome and myeloid leukaemia [27–29]. Collectively, AML1a or disease-associated AML1a-like AML1 molecules could be implicated in increased proliferation or competitiveness at key stages of hematopoietic differentiation. Further analysis of the mechanisms involved will be of therapeutic interest in the context of hematopathology and may inform attempts to expand transplantable hematopoietic stem/progenitor cells for cell therapy. Of particular interest as a stem cell source is cord blood. It is widely available through cord-blood banks, is applicable across human leukocyte antigen mismatches, and carries low risks of infectious disease transmission [61]. Despite these merits, slow engraftment and limited cell numbers contained in a given umbilical cord increases the likelihood of serious infectious complications and limits widespread application to adult patients. Modest expansion of stem and progenitor cells would be expected to circumvent these drawbacks [62], and thus *AML1a*-mediated mechanisms may therefore prove useful in the facilitation of transplantation therapeutics. Our experiments on human cord blood cells support this view. We have shown at the RNA level that *AML1a* is predominantly expressed within the primitive CD34^+^CD133^+^ and CD34^+^CD33^+^ compartments of human cord blood. Although human HSCs have not been fully characterized, these compartments are thought to contain the bulk of HSCs and multipotent progenitors [63–66]. Thus it is plausible that expansion of hematopoietic cells could be normally affected, in part, by AML1a expression by a combination of antagonism of AML1b but also through AML1a-specific pathways (see above). It should be stressed, however, that in all these compartments, at least at steady state, *AML1b* transcripts are significantly more abundant than those of *AML1a*. However, analysis of relative protein abundance in CD34^+^ versus CD34^−^ cells suggests that *AML1a* may comprise a significant fraction of total AML1 protein in the CD34^+^ compartment. Whatever the physiological role of *AML1a,* our forced expression experiments indicate that it has the capacity to enhance primitive hematopoietic cell activity in cord blood; forced expression of AML1a in primitive CD34^+^CD38^−^ cord blood-derived cells resulted in increased LTC-IC activity, increased proportions of immunophenoptypically primitive cells in long-term MS-5 stromal cultures, and most importantly, enhanced primitive cell activity in vivo assessed in the NOD-SCID mouse model. Identification and exploitation of processes that normally regulate the relative balance of *AML1a* and *AML1b* could therefore provide one strategy to potentiate the engraftment activity of human hematopoietic stem and progenitor cells. Alternatively direct introduction of AML1a protein as a trans-activating transduction-fusion, along the lines proposed for TAT-HOXB4 [19], could be considered. In any event, our results identify AML1a as one of the few molecules [19] with the capacity for increasing the engraftment potential of hematopoietic stem and progenitor cells. In summary, our findings are of interest with respect to both the physiological and pathological roles of AML1 isoforms and have clear implications for hematopoietic cell transplantation.

## Supporting Information

Figure S1 BM Cells from 5-FU-Treated Mice Were Transduced with *AML1a*-Containing or Control Retrovirus and Transplanted into Lethally Irradiated Syngeneic Hosts The following analyses were conducted 4 mo after the transplantation.
(A) FACS analysis of the primitive c-kit^+^lin^−^ (B220, CD3, CD4, CD8, CD5, Gr-1, Mac-1, and TER-119) progenitor compartment. Results are shown from the GFP^+^ and GFP^−^ fractions of a typical *AML1a* animal. GFP^+^ cells from a typical mouse receiving vector-only-transduced cells provide a control.
(B) Proportion of cells representative of different myeloid maturation stages and erythroid and lymphoid lineages in GFP^+^ BM cells from a typical vector-only or *AML1a* animal.
(C) Cytospins of GFP^+^ BM cells from a typical vector-only animal (a), GFP^−^ (b), and GFP^+^ (c) cells from a typical *AML1a* animal.
(5.5 MB PPT)

Figure S2 BM Cells from 5-FU-Treated Mice Were Transduced with *AML1a*-Containing, *AML1b*-Containing, or Control Retrovirus and Cultured under Multimyeloid Differentiation Conditions (A) Photomicrograph of typical colonies derived from control, *AML1a-,* or *AML1b*-transduced BM cells at the second round of re-plating.
(B) Photomicrographs of cells from 8-wk-old suspension cultures derived from *AML1a*-expressing colonies obtained at the third round of replating (see text for details). May-Grunwaldt-Giemsa staining of the adherent (upper image) and floating cells (lower image).
(1.0 MB PPT)

### Accession Numbers

The GenBank (http://www.ncbi.nlm.nih.gov/) accession numbers of the genes discussed in this paper are *AML1a* (D43967), *AML1b* (D43968), *AML1c* (D43969), *Aml1* (NM_009821), *GATA-1* (X17254), *SCL/TAL* (NM_003189), *TEL/ETV6* (NM_001987), and *IKAROS/IKZF* (U40462).

## Abbreviations

BM: bone marrow
BrdU: 5-bromo-2′-deoxyuridine
CAFC;: cobblestone area-forming cell
CFC: colony-forming cell
CFU: colony-forming unit
CFU-G: granulocyte-restricted CFU
CFU-GM: granulocyte/macrophage-restricted CFU
CFU-meg: CFU megakaryocyte
CFU-mix: CFU mixed lineage
CSF: colony-stimulating factor
Epo: erythropoietin
FACS: fluorescence-activated cell sorter
5-FU: 5-fluorouracil
GFP: green fluorescent protein
HSC: hematopoietic stem cell
IL: interleukin
IRES: internal ribosome entry site
KSL: c-Kit+ Sca-1+Lin−
LTC-IC: long-term culture initiating cell
meg: megakaryocyte
SCF: stem cell factor
